# A Simple, Fast, and Inexpensive Simultaneous Determination of Trace Bismuth(III) and Lead(II) in Water Samples by Adsorptive Stripping Voltammetry

**DOI:** 10.1155/2017/1486497

**Published:** 2017-08-28

**Authors:** Malgorzata Grabarczyk, Marzena Adamczyk

**Affiliations:** Department of Analytical Chemistry and Instrumental Analysis, Faculty of Chemistry, Maria Curie-Sklodowska University, 20-031 Lublin, Poland

## Abstract

A simple, fast, and inexpensive voltammetric method for the simultaneous determination of trace bismuth(III) and lead(II) using (Hg(Ag)FE) as a working electrode was optimized. For adsorptive stripping voltammetric determination of Bi(III) and Pb(II) in a single scan, the cupferron was applied as a complexing agent. Experimental conditions under which these elements can be simultaneously detected include 0.1 mol L^−1^ acetate buffer (pH = 4.6), 1 × 10^−4^ mol L^−1^ cupferron, accumulation potential −0.05 V, and accumulation time 30 s. The experiments were performed without deaeration of the solutions. The calibration graph was linear from 2 × 10^−9^ mol L^−1^ to 1 × 10^−7^ mol L^−1^ for the simultaneous presence of bismuth and lead. The detection limits for preconcentration time of 30 s were 6.7 × 10^−10^ mol L^−1^ and 8.8 × 10^−10^ mol L^−1^ for bismuth and lead, respectively. The application of this procedure was tested by analyzing certified reference material (SPS-WW1 Wastewater) and Lake Zemborzyce water (eastern areas of Poland).

## 1. Introduction

The scope for a simultaneous determination of two or more metals at ultratrace levels has always awaken interest in natural water samples analysis [1]. One of the most appropriate techniques that makes possible the determination of very low concentrations and simultaneous determination of several elements in one measurement is stripping voltammetry. Hence, in recent years, a lot of voltammetric procedures for the simultaneous determination of different metals have been reported in the literature [2–17]. As can be seen, in the case of simultaneous determination of bismuth with other elements, in the great majority, the procedures concern determination of bismuth with copper in one measurement [12–16]. As regards cadmium the simultaneous determination of this metal is connected with cadmium [2, 4–6]. We have managed to find only one paper relating to simultaneous determination bismuth and lead [17].

Bismuth and lead are among the most interesting metals which have received extensive attention in the field of trace metal analysis. There is concern about the fact that bismuth has been exploited in many various areas of industry, for instance, in the chemical and metallurgical industries, in the production of low melting alloys and thermoelectric materials, in pharmaceutical industry, in the production of cosmetics, and in semiconductor devices such as CCDs or photodiodes [18–22]. Lead is also an important metal in many kinds of industrial processes. These heavy metals are introduced into the environment from a variety of human activities such as the manufacture of batteries, metal products, leaded gasoline, paints, and stabilizers for PVC, crystal glass, and ceramic glazes [23–26]. For this reason, bismuth and lead are considered to be anthropogenic pollutants of the environment, and so the monitoring of these elements is a very important issue. There are several methods which have been used for bismuth and lead determination including inductively coupled plasma emission spectrometry or mass spectrometry [27–29], flame, and electrothermal or hydride generation atomic absorption spectrometry [30–35]. The advantages of these methods are excellent sensitivity, good selectivity, and a wide linearity range, but, on the other hand, they require expensive instruments, so they may be prohibitive to many laboratories. Moreover separation from other elements present in the sample and also the use of a preconcentration is usually necessary.

The present paper describes a simple, fast, inexpensive and at the same time very sensitive procedure for the simultaneous determination of bismuth and lead in environmental water samples. Total measurement time is of about 30 s and the analyzed sample does not require any pretreatment and is added directly in its original form to the measurement cell. The scheme is built around the adsorptive stripping voltammetric method and is based on the accumulation of Bi(III)-cupferron and Pb(II)-cupferron complexes and following its reduction via the use of an appropriate variation of potential. A single potential scan allowed us to obtain qualitative and quantitative information about the trace bismuth and lead levels. The complexes of Bi(III)-cupferron and Pb(II)-cupferron were accumulated due to their adsorption on a renewable mercury film silver based electrode (Hg(Ag)FE) used as a working electrode. The Hg(Ag)FE electrode is a viable alternative to the hanging mercury drop electrode (HMDE) because it guarantees all advantages of the mercury electrode while at the same time thanks to its construction greatly reducing toxicity.

This work was optimized by a selection of optimum conditions including the selection of the supporting electrolyte, its pH, concentration of the cupferron, and accumulation potential and time. The current study has a comparative advantage in relation to the only procedure of simultaneous determination bismuth and lead which we find in literature data [17]. These benefits are significantly lower detection limits, use of commonly available complexing agent, and above all application of more friendly for lab analysis and environment working electrode.

## 2. Experimental

### 2.1. Reagents and Solutions

A standard solution of 1 g L^−1^ of Pb(II) was obtained from Fluka (Buchs, Switzerland). Standard solutions of 1 g L^−1^ of Bi(III) and cupferron (N-nitrosophenylhydroxylamine ammonium salt) were obtained from Merck (Darmstadt, Germany). A 1 mol L^−1^ acetate buffer (pH = 4.6) was prepared from Suprapur CH_3_COOH and NaOH obtained from Merck. Triton X-100, sodium dodecyl sulfate (SDS), and cetyltrimethylammonium bromide (CTAB) were purchased from Fluka. Humic acid sodium (HA) salt was obtained from Aldrich. The river fulvic acid (FA) and natural organic matter were a standard sample obtained from the Suwannee River and purchased from the International Humic Substances Society. Amberlite XAD-7 resin and Rhamnolipid (biosurfactant) were obtained from Sigma. Amberlite XAD-7 resin was washed four times in water and dried up at the temperature of 50°C before use. The standard material SPS-WW1 Batch 111-Reference Material for Measurement of Elements in Wastewaters was obtained from Spectrapure Standards, Oslo, Norway. All solutions were prepared using triply distilled water.

### 2.2. Apparatus

Voltammetric measurements were performed with a *μ*Autolab analyzer (Utrecht, Netherlands). The three-electrode system was completed by a renewable mercury film silver based electrode (Hg(Ag)FE) as a working electrode (its construction was described in an earlier paper [36]), platinum wire as an auxiliary electrode, and an Ag/AgCl electrode as a reference electrode.

### 2.3. General Procedure

The analyzed solution or the synthetic solution (containing adequate levels of Bi(III) and Pb(II) in 0.1 mol L^−1^ nitric acid) used for optimization of procedure conditions was pipetted into the voltammetric cell. Next the acetate buffer, cupferron, and triply distilled water were introduced in order to obtain 10 mL of the solution containing 0.1 mol L^−1^ acetate buffer (pH = 4.6), 1 × 10^-4 ^mol L^−1^ cupferron. Research showed that sequence of reagents addition could be random and did not affect voltammetric signal of bismuth and lead. The solution was not deaerated.

The measurements were performed by differential pulse adsorptive stripping voltammetry. The accumulation potential −0.05 V was applied for 30 s while stirring the solution. At the end of accumulation time, the stirrer was switched off, and, after the equilibration time of 5 s, a differential pulse voltammogram was recorded, while the potential was scanned from −0.05 V to −0.7 V. The scan rate and pulse height were 20 mV s^−1^ and −50 mV, respectively.

## 3. Results and Discussion

### 3.1. Conditions for Simultaneous Determination of Bi(III) and Pb(II)

Preliminary works showed that cupferron was a suitable complexing agent for the determination of Bi(III) and Pb(II) when an acetate buffer was used as a supporting electrolyte [37, 38]. Cupferron is commonly used in adsorptive voltammetric procedures as complexing agent, mainly applied under acidic conditions [9–11, 37–42]. The effect of pH of the buffer on the simultaneous determination of bismuth and lead was studied in the range from 3 to 6. The acetate buffer and cupferron concentrations were 0.1 mol L^−1^ and 1 × 10^-4 ^mol L^−1^, respectively, and the concentration of metal ions was 2 × 10^-8 ^mol L^−1^, the deposition potential −0.05 V, and the deposition time 30 s. The main factors which were taken into consideration were height and shape of bismuth and lead peaks and separation of the peaks. In the case of bismuth a variation of the pH in the whole verified range did not cause any significant change of the voltammetric signal. In the case of lead the signal remained constant in the range from 4 to 6, for lower values it gradually decreased. Taking into account the above details as well as good buffering capacity, a pH value of 4.6 was chosen for the whole study.

**Figure 1:**
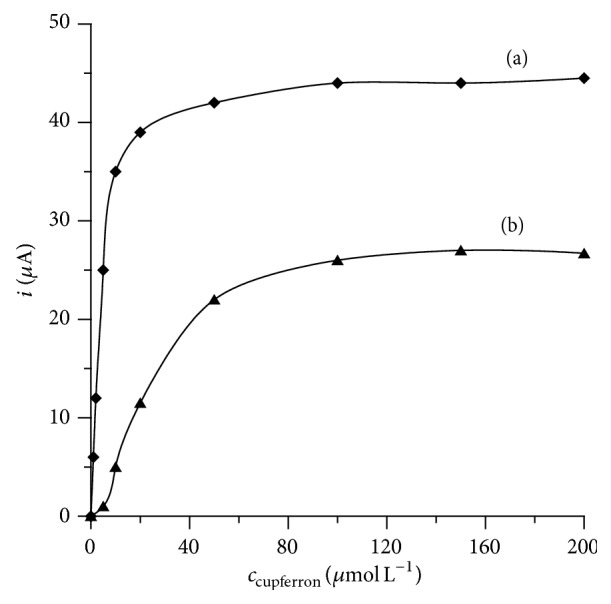
The influence of cupferron concentration on the 2 × 10^−8^ mol L^−1^ Bi(III) (a) and 2 × 10^−8^ mol L^−1^ Pb(II) (b) peak current. Accumulation potential −0.05 V and accumulation time 30 s. pH acetate buffer equal to 4.5.

The dependence between the Bi(III)-cupferron and Pb(II)-cupferron peak currents on the complexing agent concentration was tested. The concentration of cupferron ranged from 1 × 10^-6 ^mol L^−1^ to 5 × 10^-4 ^mol L^−1^ in the presence of 2 × 10^-8 ^mol L^−1^ of each of metal ions and 0.1 mol L^−1^ acetate buffer (pH = 4.6). The obtained results showed that the peak currents of both Bi(III)-cupferron and Pb(II)-cupferron increase with the increase of the complexing agent concentration up to 1 × 10^-4 ^mol L^−1^ (Figure 1).

**Figure 2:**
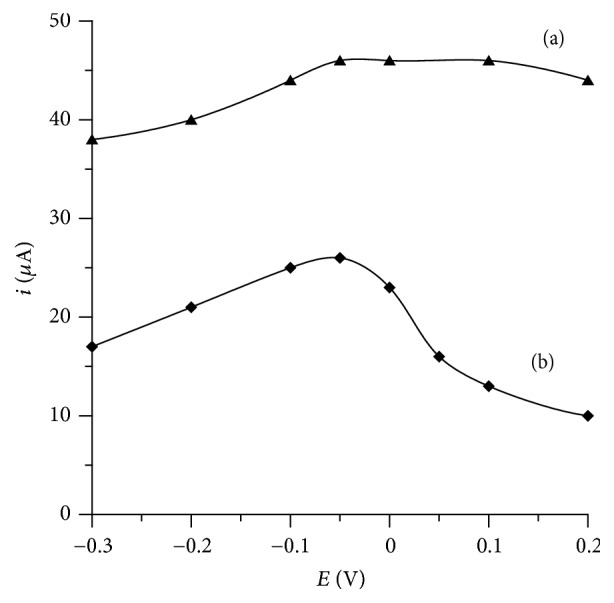
The influence of accumulation potential on the 2 × 10^−8^ mol L^−1^ Bi(III) (a) and 2 × 10^−8^ mol L^−1^ Pb(II) (b) peak current. Concentration of cupferron 1 × 10^−4^ mol L^−1^ and acetate buffer 0.1 mol L^−1^ (pH = 4.5). Accumulation time 30 s.

The effect of the accumulation potential on the peak currents over the range of 0.2 V to −0.3 V for 2 × 10^-8 ^mol L^−1^ Bi(III) and Pb(II) showed that the peak current for Bi(III) was almost stable in the whole range with a slight tendency to decrease for a more negative potential and the peak current for Pb(II) attained a maximum at the potential of about −0.05 V. So the accumulation potential equal to −0.05 V was selected for further experiments, as the most suitable for both metal ions (Figure 2).

**Figure 3:**
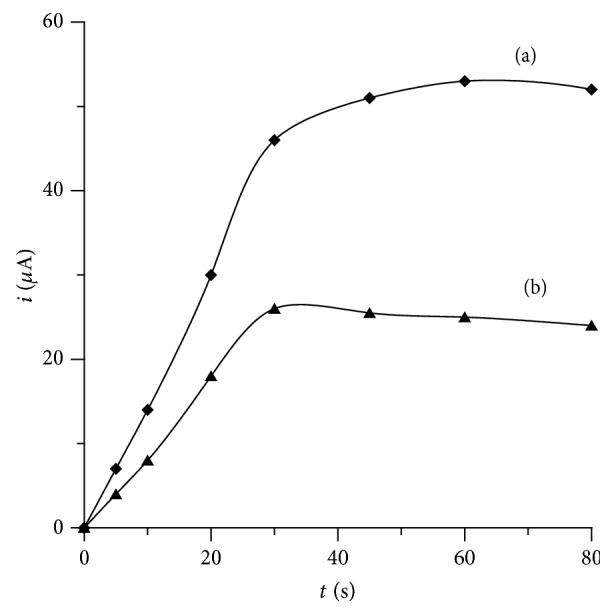
The influence of accumulation time on the 2 × 10^−8^ mol L^−1^ Bi(III) (a) and 2 × 10^−8^ mol L^−1^ Pb(II) (b) peak current. Concentration of cupferron 1 × 10^−4^ mol L^−1^ and acetate buffer 0.1 mol L^−1^ (pH = 4.5). Accumulation potential −0.05 V.

The effect of accumulation time on peak currents was examined in the 0–80 s range, while other standard measuring conditions remained constant as described above. The values of the voltammetric peak currents increased when accumulation time was prolonged to 30 s and 60 s for lead and bismuth, respectively (Figure 3).

### 3.2. Linear Ranges and Detection Limits

Linear ranges and detection limits for the simultaneous determination of bismuth and lead were evaluated under the selected conditions: 0.1 mol L^−1^ acetic buffer (pH = 4.6), 1 × 10^-4 ^mol L^−1^ cupferron, accumulation potential −0.05 V, and accumulation time 30 s. Linear calibration graphs were obtained in the concentration range of 2 × 10^-9 ^mol L^−1^ to 1 × 10^-7 ^mol L^−1^ for the simultaneous presence of Bi(III) and Pb(II) in the solution. They obeyed the following calibration equations: *y* = 1.97*x* + 3.05 (for Bi(III)) with the linear correlation coefficient *r* = 0.996, and *y* = 1.20*x* + 1.41 (for Pb(II)) with the linear correlation coefficient *r* = 0.995, where *y* and *x* are the peak current (*μ*A) and concentration (nmol L^−1^), respectively. The detection limits estimated from three times the standard deviation of low Bi(III) and Pb(II) concentrations and accumulation time 30 s were about 6.7 × 10^-10 ^mol L^−1^ and 8.8 × 10^-10 ^mol L^−1^, respectively. The relative standard deviations (RSD) from six determinations at the concentrations 5 × 10^-9 ^mol L^−1^ of Bi(III) and Pb(II) were 3.9% and 5.0%, respectively.

### 3.3. Interference of Organic Compounds

**Figure 4:**
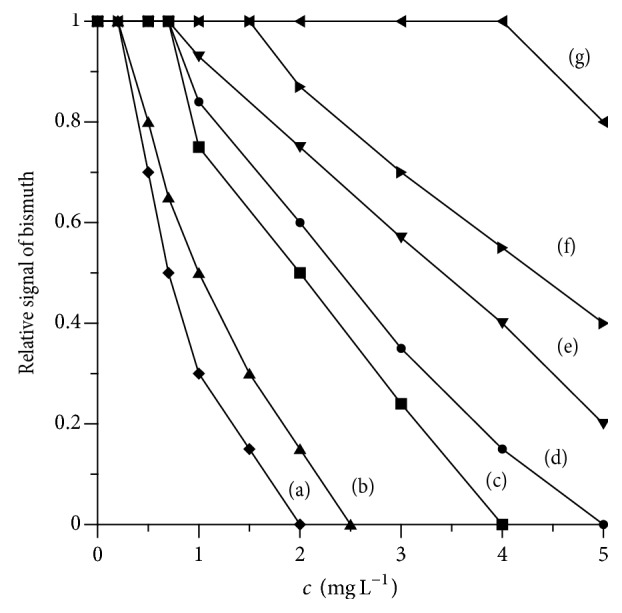
The influence of Triton X-100 (a), Rhamnolipid (b), HA (c), CTAB (d), FA (e), NOM, (f) and SDS (g) on 2 × 10^−8^ mol L^−1^ Bi(III) peak current. Concentration of cupferron 1 × 10^−4^ mol L^−1^ and acetate buffer 0.1 mol L^−1^ (pH = 4.5). Accumulation potential −0.05 V and accumulation time 30 s.

The aim of the proposed procedure was to analyze environmental water samples, so the influence of organic compounds present in natural samples such as surface active substances and humic substances should be taken into account. As it was proved in the previous procedures using HMDE as a working electrode and cupferron as a complexing agent, the organic compounds seriously suppress particularly the voltammetric signal of bismuth [37]. In the case of lead, the interference of organic substances was lower [38]. In the proposed procedure using Hg(Ag)FE as a working electrode, the influence of surface active substances, such as Triton X-100 (nonionic surfactant), sodium dodecyl sulfate (SDS, anionic surfactant), cetyltrimethylammonium bromide (CTAB, cationic surfactant), and Rhamnolipid (biosurfactant) and humic substances, such as humic acids (HA), fulvic acids (FA), and natural organic matter (NOM), was examined. No significant effect of surface active substances on the peak of lead was observed, their concentrations being no less than 5 ppm did not interfere. In the case of humic substances, the permitted levels reached 2 ppm. The influence of organic substances on the bismuth signal was more noticeable and is presented in Figure 4. The organic substances clearly reduced the voltammetric signal, particularly in the case of bismuth. In order to analyze natural samples rich in organic matter the preliminary mixing of the analyzed sample with Amberlite XAD-7 resin is recommended as an efficient and well known [39–42] way to eliminate organic interferences.

### 3.4. Analysis of Certified Reference Material

Much to our regret, the certified reference material with environmental water matrices containing both bismuth and lead at low concentrations was unreachable. So the proposed method was tested by analysis of the certified reference material SPS-WW1 Wastewater (Batch 111) containing trace elements such as Al, As, Cd, Co, Cr, Cu, Fe, Mn, Ni, P, Pb, V, and Zn with the certified value 100.0 ± 0.5 ng mL^−1^. As regards bismuth, it was additionally spiked with the same concentration. Three replicate determinations using the standard addition method were performed giving the values 96.2 ± 0.6 ng mL^−1^ and 94.8 ± 0.4 ng mL^−1^ for bismuth and lead, respectively. The measurements were performed using standard conditions but given the fact that the solution of the certified reference material SPS-WW1 contains nitric acid, a proper quantity of sodium hydroxide was additionally added in order to neutralize pH.

**Figure 5:**
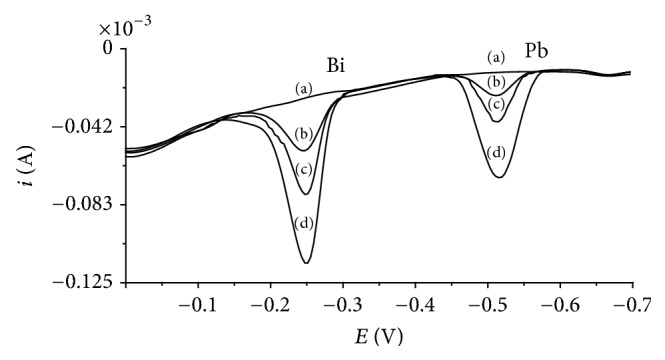
Differential pulse voltammograms obtained in the course of the Bi(III) and Pb(II) determination in Lake Zemborzyce water: (a) Lake Zemborzyce water diluted two times; (b) as (a) + 1 × 10^−8^ mol L^−1^ Bi(III) and Pb(II); (c) as (a) + 2 × 10^−8^ mol L^−1^ Bi(III) and Pb(II); (d) as (a) + 4 × 10^−8^ mol L^−1^ Bi(III) and Pb(II). Concentration of cupferron 1 × 10^−4^ mol L^−1^ and acetate buffer 0.1 mol L^−1^ (pH = 4.5). Accumulation potential −0.05 V and accumulation time 30 s.

Additional in order to demonstrate the applicability and reliability of the presented procedure for real water samples, Lake Zemborzyce (eastern areas of Poland) water was analyzed. The voltammograms recorded for this one did not exhibit any signals of Bi(III) and Pb(II), so the analyzed sample was spiked with Bi(III) and Pb(II). Three replicate determinations using the standard addition method gave the average recovery values between 95.5 and 98.6% for Bi(III) with relative standard deviation between 6.0 and 6.7% and 94.2 and 97.0% for Pb(II) with relative standard deviation between 5.8 and 7.3%. The typical voltammograms obtained in the course of this analysis are presented in Figure 5.

## 4. Conclusion

This work presents a new, simple, fast, and inexpensive procedure for the simultaneous determination of bismuth and lead ions. The adsorptive stripping voltammetry using cupferron as a complexing agent and renewable mercury film silver based electrode as a working electrode was successfully employed. The method offers low detection limits equal to 6.7 × 10^−10^ mol L^−1^ and 8.8 × 10^-10 ^mol L^−1^ for Bi(III) and Pb(II), respectively, with good relative standard deviations below 5%. The validity of the method was very successful, as it was shown by the analysis of wastewater certified reference material SPS-WW1. In case that the analyzed sample contains a large concentration of organic compounds the preliminary mixing with Amberlite XAD-7 resin should be performed. An important advantage of the proposed procedure is the fact that no deaeration of the solution is necessary, which makes it easy to use under laboratory and field conditions.
